# A Case of Infrapatellar Fat Pad Ganglion of the Knee

**DOI:** 10.2174/1874325001711011142

**Published:** 2017-10-31

**Authors:** Tsuneari Takahashi, Masashi Kimura, Takashi Ohsawa, Naoki Yamaguchi, Katsushi Takeshita

**Affiliations:** 1 Department of Orthopedic Surgery, Jichi Medical University, Shimotsuke, Japan; 2 Gunma Sports Medicine Research Center, Zenshukai Hospital, Maebashi, Japan; 3 Department of Orthopedic Surgery, Faculty of Medicine, Gunma University, Maebashi, Japan; 4 The Center for Graduate Medical Education, Jichi Medical University Hospital, Shimotsuke, Japan

**Keywords:** Knee, Meniscus, Ganglion, Arthroscopy, Infrapatellar fat pad

## Abstract

**Introduction:**

A ganglion cyst can induce symptoms around the knee and should be considered as an intra-articular mass in differential diagnosis.

**Case Presentation:**

A 22-year-old female presented with a persistent medial knee joint pain in her left knee for 2 years. There was soft tissue swelling on the anteromedial aspect of the infrapatellar region on her left knee. Lachman and McMurray tests were negative. MRI showed a multilobular cyst in the infrapatellar fat pad with T1 low intensity and T2 STIR high intensity. The cyst was not attached to either meniscus. ACL and PCL looked normal.

During surgery, the cyst was found to arise from the intra-patellar fat pad and was not attached to the menisci or synovium. The cyst was completely resected.

Histological findings showed a multilobular cyst with a glassy fibrous tissue wall and clear jelly-like consistency, confirming the diagnosis of a ganglion. The patient recovered asymptomatically and has been without recurrence 7 years postoperatively.

**Conclusion:**

Differential diagnoses of an infrapatellar swelling are a meniscal cyst, synovial cyst, or ganglion. Most cases of cysts around the knee generate from fluid collection through meniscal tears. A ganglion cyst is a synovium-lined structure and is common around the wrist joint, but rare in the knee joint. A ganglion cyst in the knee joint often arises from ACL or PCL, but rarely arises from the infrapatellar fat pad. A ganglion cyst is one of the differential diagnoses of parameniscal cysts around the knee. We recommended an open resection with arthroscopic examination.

## 1 INTRODUCTION

Cysts around the knee joint are common. Most such cases involve fluid collection through meniscal tears [1]. Sometimes, a parameniscal cyst induces symptoms around the knee, such as pain or sense of incongruity. A ganglion cyst also induces symptoms around the knee and should be considered as an intra-articular mass in a differential diagnosis.

## 2 CASE PRESENTATION

**Fig. (1):**
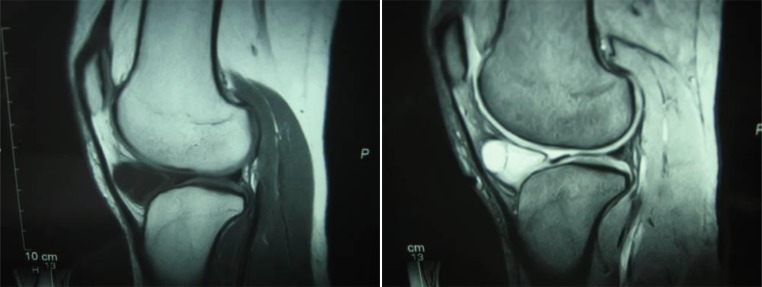
MRI showed a multilobular cyst in the infrapatellar fat pad with T1 low intensity and T2 STIR high intensity. The cyst was not attached to either menisci.

A 22-year-old female presented with persistent medial knee joint pain in her left knee for 2 years. She had no history of trauma to her left knee. On first examination at our department, she looked healthy but complained of tenderness in the medial knee joint. There was soft tissue swelling on the anteromedial aspect of the infrapatellar region on her left knee. She had a full range of motion and other physical findings, such as the Lachman and McMurray tests, were negative. An X-Ray showed no bony changes induced by osteoarthritis. MRI showed a multilobular cyst in the infrapatellar fat pad with T1 low intensity and T2 STIR high intensity (Fig. **1**). The cyst was not attached to either menisci. ACL and PCL looked normal.

## 3 OPERATIVE FINDINGS

**Fig. (2):**
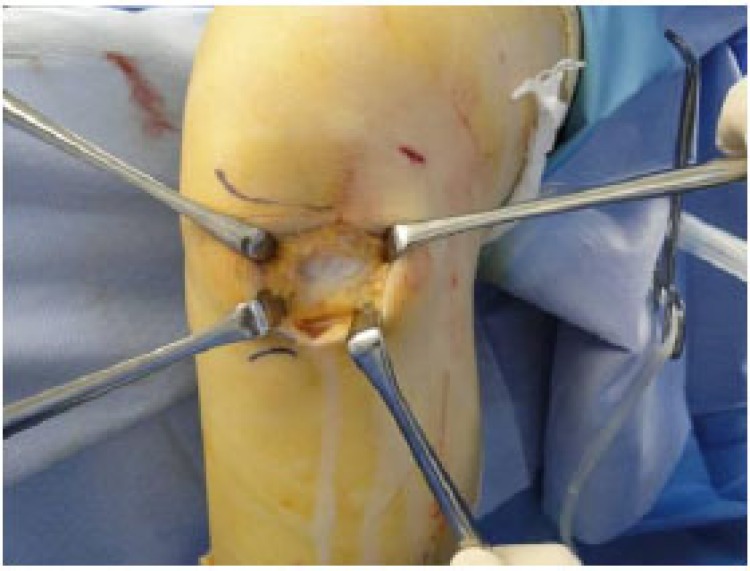
We made a 5-cm lateral parapatellar incision to approach the cyst, which was successfully resected.

**Fig. (3):**
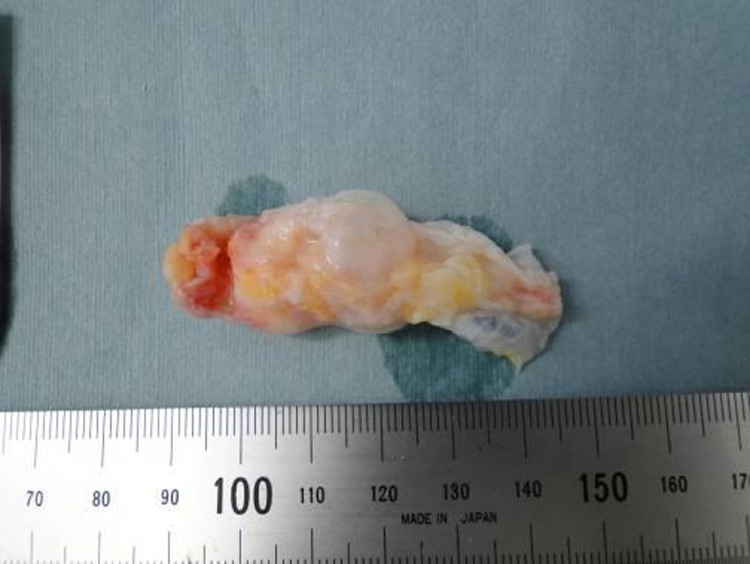
The multilobular cyst, which had a size of 5.5 × 2.0 × 1.0 cm, was completely resected.

During surgery, arthroscopy showed no tears in either menisci, ACL or PCL, nor was the cyst found. Because of the arthroscopic findings, we made a 5-cm lateral parapatellar incision to approach the cyst (Fig. **2**). The cyst arose from the intra-patellar fat pad and was not attached to menisci or synovium. The cyst was completely resected; its size was 5.5 × 2.0 × 1.0 cm (Fig. **3**). After resection, the wound was closed in layers.

## 4 HISTOLOGICAL EVALUATION

**Fig. (4):**
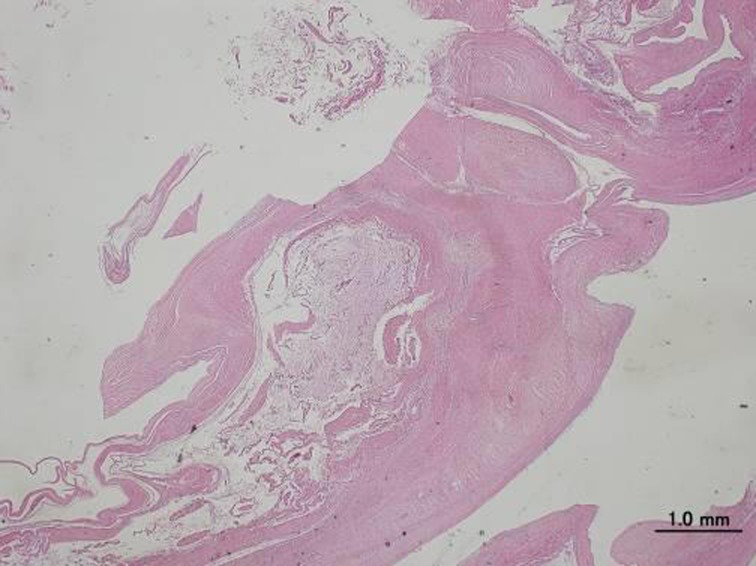
HE staining showed a multilobular cyst with glassy fibrous tissue wall and clear jelly-like consistency. There were no coverage cells inside the wall and these findings confirmed the diagnosis of a ganglion. The length of the bar is 1.0 mm.

Histological findings showed a multilobular cyst with a glassy fibrous tissue wall and clear jelly-like consistency. There were no coverage cells inside the wall, which confirmed the diagnosis of a ganglion (Fig. **4**). The patient recovered asymptomatically and has been without recurrence 7 years postoperatively.

## 5 DISCUSSION

Fat pad of the knee is affected by a variety of tumours and tumour-like conditions [2]. Differential diagnoses of infrapatellar swelling are osteochondroma, localised pigmented villonodular synovitis, meniscal cyst, synovial cyst, or ganglion [3]. The commonest presenting symptom was anterior knee pain. We sometimes experience cysts around the knee, such as intrameniscal, parameniscal, or synovial cysts, and meniscocapsular separation [4]. Most cases of cysts around the knee are generated from fluid collection through meniscal tears. A ganglion cyst is a synovium-lined structure and is common around the wrist joint, but it is rare in the knee joint. A ganglion cyst in the knee joint often arises from ACL or PCL [5], but rarely arises from the infrapatellar fat pad. Sugiura *et al.* reported the ganglion cyst arising from the infrapatellar fat pad of 10-year-old boy [6]. Sloane *et al*. reported the case of 41-year-old female who presented with a gradually worsening anterior knee pain, swelling and inability to flex the knee. MRI revealed a large intra-articular cystic swelling anterior to ACL, extending into the infrapatellar fat pad [5]. In a study by Krudwig *et al.* [7], 76 cases of ganglion cysts were found in 8000 knee arthroscopic examinations. Most of them arose from ACL or PCL. In contrast, only three cases arose from the infrapatellar fat pad. Mine *et al.* [8] and Yilmaz *et al.* [9] have reported on cases of ganglion cysts in the infrapatellar fat pad of the knee. In their studies, the patient presented with swelling around the knee joint. Trompeter *et al.* reported on an acute locked knee induced by a ganglion cyst in the infrapatellar fat pad of the knee [10]. Schmitz *et al.* reported a case of a ganglion that had invaded the infrapatellar fat pad, but it was not the infrapatellar fat pad the ganglion had originated from but the medial meniscus [11]. MRI is an important modality for diagnosing ganglion cysts [12]. MRI findings are T1 low intensity and T2 high intensity for all lesions [13]. In case of ganglion cysts in the infrapatellar fat pad of the knee, it is difficult to reach the cyst arthroscopically although Yang *et al*. reported an endoscopic resection of a ganglion cyst in an infrapatellar fat pad extending into the subcutaneous layer [14]. Bisicchia *et al.* reported the recurrence of infrapatellar fat pad cysts after ultrasound-guided aspiration [15]. Saha *et al.* described that subcutaneous extension of infrapatellar fat pad ganglion may lead to incomplete arthroscopic resection and leaving behind the residual tissue which may cause recurrence [16]. Nikolopoulos *et al.* described that when there is a large ganglion, treatment should be an open and thorough resection [17]. Therefore, we should perform open surgery in case a ganglion cyst in the infrapatellar fat pad of the knee is suspected because there are no reports in the literature on recurrence following open resection [13].

## CONCLUSION

We experienced a case of a ganglion cyst developed in the infrapatellar fat pad of the knee. The patient completely recovered after open resection without arthroscopic treatment. Ganglion cyst is one of the differential diagnoses of a parameniscal cyst around the knee. We recommend not an arthroscopic resection but an open resection with arthroscopic examination to avoid recurrence.

## References

[1] Cowden C.H., Barber F.A. (2014). Meniscal cysts: treatment options and algorithm.. J. Knee Surg..

[2] Dean B.J., Reed D.W., Matthews J.J., Pandit H., McNally E., Athanasou N.A., Gibbons C.L. (2011). The management of solitary tumours of Hoffa’s fat pad.. Knee.

[3] Nouri H., Ben Hmida F., Ouertatani M., Bouaziz M., Abid L., Jaafoura H., Zehi K., Mestiri M. (2010). Tumour-like lesions of the infrapatellar fat pad.. Knee Surg. Sports Traumatol. Arthrosc..

[4] Mink J.H., Deutsch A.L. (1989). Magnetic resonance imaging of the knee.. Clin. Orthop. Relat. Res..

[5] Sloane J., Gulati V., Penna S., Pastides P., Baghla D.P. (2010). Large intra-articular anterior cruciate ligament ganglion cyst, presenting with inability to flex the knee.. Case Rep. Med..

[6] Sugiura K., Suzue N., Matsuura T., Hamada D., Goto T., Takata Y., Sairyo K. (2015). Ganglion cyst arising from the infrapatellar fat pad in a child.. J. Med. Invest..

[7] Krudwig W.K., Schulte K.K., Heinemann C. (2004). Intra-articular ganglion cysts of the knee joint: A report of 85 cases and review of the literature.. Knee Surg. Sports Traumatol. Arthrosc..

[8] Mine T., Ihara K., Tanaka H., Taguchi T., Azuma E., Tanigawa Y., Kawai S. (2003). A giant ganglion cyst that developed in the infrapatellar fat and partly extended into the knee joint.. Arthroscopy.

[9] Yilmaz E., Karakurt L., Ozercan I., Ozdemir H. (2004). A ganglion cyst that developed from the infrapatellar fat pad of the knee.. Arthroscopy.

[10] Trompeter A., Servant C. (2009). Case report. An unusual cause of a patient presenting with an acutely locked knee: multiple benign fat pad cysts.. Arch. Orthop. Trauma Surg..

[11] Schmitz M.C., Schaefer B., Bruns J. (1996). A ganglion of the anterior horn of the medial meniscus invading the infrapatellar fat pad. Case report.. Knee Surg. Sports Traumatol. Arthrosc..

[12] Ghate S.D., Deokar B.N., Samant A.V., Kale S.P. (2012). Tumor like swellings arising from Hoffa’s fat pad: A report of three patients.. Indian J. Orthop..

[13] Amin M., Torreggiani W., Sparkes J. (2008). Infrapatellar ganglion that developed from infrapatellar fat and had minimal intraarticular extension.. Knee Surg. Sports Traumatol. Arthrosc..

[14] Yang J.H., Kim T.S., Lim H.C., Kim H.J., Kim Y.J., Oh C.H., Yoon J.R. (2012). Endoscopic excision of a ganglion cyst in an infrapatellar fat pad extending into the subcutaneous layer.. J. Orthop. Sci..

[15] Bisicchia S., Savarese E. (2013). Infra-patellar fat pad cysts: a case report and review of the literature.. Muscles Ligaments Tendons J..

[16] Saha P., Bandyopadhyay U., Mukhopadhyay A.S., Kundu S., Mandal S. (2015). Ganglion Cyst of Knee from Hoffa’s Fat Pad Protruding Anterolaterally Through Retinacular Rent: A Case Report.. J Orthop Case Rep.

[17] Nikolopoulos I., Krinas G., Kipriadis D., Ilias A., Giannakopoulos A., Kalos S. (2011). Large infrapatellar ganglionic cyst of the knee fat pad: a case report and review of the literature.. J. Med. Case Reports.

